# Impact of ischemic preconditioning combined with aerobic exercise on 24-h ambulatory blood pressure in men with prehypertension and stage 1 hypertension

**DOI:** 10.3389/fphys.2024.1495648

**Published:** 2024-11-07

**Authors:** Min-Hyeok Jang, Dae-Hwan Kim, Jean-Hee Han, Seok-Ho Kim, Jung-Hyun Kim

**Affiliations:** ^1^ Department of Physical Education, General Graduate School, Kyung Hee University, Yongin, Republic of Korea; ^2^ Department of Sports Medicine, Kyung Hee University, Yongin, Republic of Korea

**Keywords:** ischemic preconditioning, hypertension, post-exercise hypotension, ambulatory blood pressure, aerobic exercise

## Abstract

**Introduction:**

A single bout of aerobic exercise is known to induce a temporary reduction in post-exercise blood pressure termed post-exercise hypotension (PEH). Meanwhile, an ischemic preconditioning (IPC), a series of short ischemia-reperfusion intervention, has also shown antihypertensive effects showing a potential nonpharmacologic intervention for hypertension. While the acute BP reduction effects of aerobic exercise and IPC are individually well-investigated, it remains unclear if combining both interventions has an additive effect on PEH.

**Methods:**

A total of twelve pre- or hypertensive men (six prehypertension, six stage 1 hypertension) underwent either 30 min of aerobic exercise at 50% VO_2peak_ (CON) or IPC before exercise, in a counterbalanced order. IPC involved inflating cuffs on both thighs to 200 mmHg for 5 min, alternating between right and left thighs for three cycles, totaling 30 min. Brachial BP was measured during exercise and 1-h post-exercise recovery whereas muscle oxygen saturation (SmO_2_) from the rectus femoris was monitored using NIRs during exercise and recovery. Heart rate variability (HRV) and baroreflex sensitivity (BRS) together with a head-up tilt test (at 0 and 50°) were measured at the pre-test, post-test, and 24-h post-test. After the completion of each experiment, 24-h ambulatory blood pressure (ABP) was monitored to assess post-exercise hypotension within a 24-h window.

**Results:**

BP and heart rate responses during exercise and 1-h recovery did not differ between conditions while SmO_2_ was significantly elevated during exercise in IPC (*p* = 0.004). There was no difference in HRV and supine BRS. However, significantly reduced titled BRS after exercise was found in CON while IPC preserved BRS similar to pre-exercise value, extending to 24-h post period (*p* = 0.047). ABP monitoring revealed a significant reduction in systolic BP during sleep in IPC compared to CON (*p* = 0.046).

**Conclusion:**

The present findings suggest that IPC with a single session of aerobic exercise results in a notable decrease in systolic ABP, particularly during sleep, compared to aerobic exercise alone. This supplementary antihypertensive effect was associated with a sustained BRS, persisting up to 24 h in contrast to the significant decrease observed in CON. Future studies are warranted to investigate long-term adaptations to IPC.

## 1 Introduction

Hypertension, defined as systolic blood pressure (SBP) ≥ 130 mmHg and/or diastolic blood pressure (DBP) ≥ 80 mmHg is a prevalent worldwide, fatal medical condition with a high probability of leading to ischemic heart disease, stroke, and end-stage renal disease (WHO, 2021). Therefore, continuous BP monitoring is important not only for people with hypertension but also for those in the phase of elevated BP (Flack and Adekola, 2020). In addition to traditional hypertension treatment such as medication and exercise, ischemic preconditioning (IPC), a series of short ischemia-reperfusion interventions in faraway tissue or organ (Przyklenk et al., 1993), is emerging as a non-invasive, universally applicable, and time-efficient alternative treatment which operates through the improvement of endothelial function (Kharbanda, Nielsen and Redington, 2009; Kim et al., 2020). For instance, a single bout of IPC not only showed cardioprotective effects such as improved left ventricular pressure and end-diastolic pressure in spontaneously hypertensive young rats (Farkašová et al., 2023), but also significantly decreased peripheral/central systolic BP and mean arterial pressure (MAP) in patients with peripheral artery disease 24 h after digital subtraction angiography (Kuusik et al., 2019). Further, Madias (2011) and Zagidullin et al. (2016) reported the acute effect of IPC on BP in normotensive subjects and patients with angina pectoris, respectively. In this manner, a single bout of IPC has demonstrated effectiveness in alleviating BP in patients with various diseases, consistent with the results of our previous study targeting obese young males (Jang et al., 2023).

IPC has also been shown to significantly enhance exercise performance in healthy subjects (De Groot et al., 2010; Caru et al., 2016), athletes (Cruz et al., 2016), and patients with diseases (Chotiyarnwong et al., 2020). Such ergogenic effects may be associated with improved mitochondrial activation in local skeletal muscle (Jeffries et al., 2018; Kido et al., 2015; Tanaka et al., 2016) and acceleration of systemic oxygen extraction during exercise (Kido et al., 2018). This resulted in immediate performance improvements, such as improved power output and maximum oxygen intake during exercise in healthy subjects (De Groot et al., 2010), improved mean power output with increased skeletal muscle activation in cyclists (Cruz et al., 2016), and an improved 6-min walking test results in patients with multiple sclerosis (Chotiyarnwong et al., 2020). Moreover, IPC also demonstrated notable effects during the post-exercise recovery period. For example, it prevented the reduction in flow-mediated dilation after high-intensity running (Bailey et al., 2012) and shortened the QT interval in healthy subjects during exercise and recovery (Caru et al., 2016) suggesting the shortened action potential duration via the activation of protein kinase C. Interestingly, Beaven et al. (2012) and Seeger et al. (2017) delineated that these performance improvements could persist even 24 h after exercise.

The effectiveness of regular exercise and/or physical activity on BP reduction is widely accepted. Further, a single bout of exercise could lead to a temporary decrease in BP termed post-exercise hypotension (PEH) (Fitzgerald, 1981). While some controversies exist regarding the degree of PEH and its sustained impact under free-living conditions, numerous previous findings suggested that a single session of moderate-intensity exercise leads to PEH in diverse populations with a greater degree observed in hypertensive individuals. For example, a moderate-intensity continuous cycling exercise reduced BP (SBP: 6.3 ± 1.3 mmHg, DBP: 1.8 ± 1.0 mmHg) in healthy adults for 120 min (Keese et al., 2011). Others reported significantly reduced 24-h ambulatory blood pressure (ABP) in medicated hypertensives (Ciolac et al., 2009) and in prehypertensive individuals with type 2 diabetes, especially during sleeping (de Morais et al., 2015). In elderly people with essential hypertension, moderate-intensity treadmill exercise lowered both systolic and diastolic BP for 1 h after aerobic exercise and significantly lessened DBP during the daytime (Ferrari et al., 2017). Taken all together, a single bout of exercise leads to the mitigation of high BP to some extent and is considered to be an effective antihypertensive strategy that benefits from an acute exercise bout.

There are various non-pharmacological interventions aimed at mitigating high BP, and some have attempted to utilize these auxiliary methods in conjunction with exercise to better induce the lowering effects of BP. For example, a single session of endurance exercise followed by sauna in prehypertensive men showed a greater reduction in SBP than exercise alone, and this effect remained at 24-h follow-up (Rissanen et al., 2020). Besides, low-intensity aerobic exercise with blood flow restriction similar to IPC has also been reported to be as effective as high-intensity exercise in lowering BP (Barili et al., 2018). However, exercise with blood flow restriction causes a rapid increase in MAP during exercise, leading to cardiac overload (Thomas et al., 2018), which may be a limitation for patients with underlying cardiovascular disease. IPC as a means of addressing this impediment, when combined with resistance exercise, brought about greater and longer BP reduction in trained normotensive individuals compared to the resistance exercise alone (Panza et al., 2020). On top of that, IPC combined with aerobic exercise promoted recovery after exercise at intensities below lactate threshold in endurance runners (Sabino-Carvalho et al., 2019). However, this study was conducted with a focus on exercise performance rather than health aspects.

Although the acute BP reduction effects of IPC and aerobic exercise individually are well investigated, it is still unclear whether combining both intervention modalities would have an additive effect on PEH. Therefore, the purpose of the present study was to investigate whether combining aerobic exercise and IPC brings out a further enhancement in PEH. We hypothesized that: 1) a single bout of aerobic exercise accompanied by IPC would induce PEH to a greater extent and 2) the temporal enhancement of cardiovagal baroreflex sensitivity would contribute to this exaggerated PEH.

## 2 Materials and methods

### 2.1 Ethical approval

The present study was conducted after the approval from the Institutional Review Board at Kyung Hee University (KHGIRB-21–531) and conformed to the standard set by the Declaration of Helsinki. All participants provided written informed consent before their study participation.

### 2.2 Participants

A total of 12 male participants (6 elevated blood pressure, 6 stage 1 hypertension) who were previously diagnosed as prehypertensive or stage 1 hypertension, but currently not taking antihypertensive medication were recruited for the present study. All participants completed a medical screening questionnaire and those who reported a presence of cardiovascular disease or musculoskeletal disorder were excluded from the study. Due to the hormonal fluctuation that may potentially influence vascular outcomes, female participants were excluded. At least 24 h before the scheduled visits, they were instructed to abstain from alcohol/caffeine consumption and strenuous physical activity.

### 2.3 Experimental procedure

All participants visited the laboratory five times at one-week intervals between familiarization and experimental sessions. At the first visit, the participants underwent health screening, demographic measurements, and experimental familiarization. The experimental familiarization involved becoming accustomed to the lab environment, study procedures, and measurement equipment to ensure proper commencement of the experiment and to control for emotional effects, such as anxiety, that could influence the study outcomes. To confirm participant eligibility, brachial BP was measured triplicate in the non-dominant arm in a sitting position using a digital sphygmomanometer (BP742N, OMRON Corporation, Kyoto, Japan). Further, participants underwent a graded exercise test on a cycle ergometer (Corival CPET, Lode B.V., Groningen, Netherlands) with a constant pedal rate of 50 RPM. The workload was initially set at 50 W and was gradually increased by 25 W per minute until the participants reached volitional exhaustion., A metabolic system (Quark CPET, COSMED, Rome, Italy) was used to determine their peak oxygen uptake (VO_2peak_) throughout the test and subsequently relative exercise intensity for experimental intervention. The test was considered valid if it met at least two of the following criteria: 1) a plateau in VO_2_ despite an increase in workload, 2) a respiratory exchange ratio greater than 1.10, and/or 3) the participant achieving 85%–90% of the age-predicted maximum heart rate.

For the remaining four visits the participants visited the laboratory two consecutive days for exercise intervention and 24-h post-test measurements. For exercise intervention, the participants underwent either a bout of cycling exercise (CON) or a bout of cycling exercise accompanied by IPC intervention (IPC) which were performed in a counterbalanced order interspersed by 1 week of washout period. All experiments were conducted in the morning between 07:00 and 12:00 in a laboratory comfortably maintained at 23°C with a relative humidity of around 50%.

**FIGURE 1:**
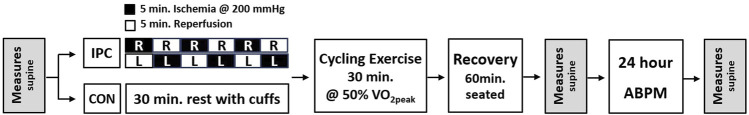
The schematic view of the study procedure.

Upon their arrival at the laboratory for experimental participation, the participants wore T-shirts, shorts, and athletic shoes. Then, they were instrumented with measurement sensors followed by 15 min of rest in a supine position for pre-test measurements after which experimental intervention commenced. For IPC, a contoured inflation cuff, connected with a rapid cuff inflation system (E20, D.E. Hokanson, Inc., Washington, United States), was placed on both the right and left distal thighs while participants rested on a cycle ergometer. The right thigh was initially occluded at an intensity of 200 mmHg for 5 min and then deflated immediately followed by alternating occlusion and reperfusion of the left thigh for 5 min each. This complete IPC cycle was repeated three times, resulting in a total procedure duration of 30 min. For CON, the participants rested on the cycle ergometer with the same cuffs placed remaining deflated on both thighs. After the completion of either IPC or CON intervention, the participants commenced cycling exercise at the pre-determined 50% maximal exercise intensity constantly maintaining greater than 50 RPM for 30 min. The mean exercise intensity was 124 W. After completing the exercise, the participants were taken out of the cycle and rested in a chair for 60 min, followed by post-test measurements in a supine position. Once all test procedures were completed, participants were equipped with an ambulatory blood pressure monitor and then returned home. After 24 h, they revisited the laboratory to return the monitor and undergo the same pre/post-test measurements. The schematic view of the study procedure is presented in Figure 1.

### 2.4 Blood pressure

Baseline, during exercise (12–21–30th minutes), and 1-h recovery (12–24–36–48–60th minutes) BP were measured on a brachial artery of the non-dominant arm using an automated BP monitor (Tango M2, SunTech Medical, Inc., North Carolina, United States) in the seated position. BP measurements were duplicated and expressed as mean SBP, DBP, and MAP.

### 2.5 Muscle oxygen saturation

Muscle oxygen saturation (SmO_2_) was measured to assess microvascular oxygenation using near-infrared spectroscopy (NIRS) (Moxy5, Fortiori Design LLC, Minnesota, United States). The NIRS sensor was affixed to the rectus femoris at the upper third of the right femur for continuous monitoring during exercise and 1-h recovery period. One-minute average values recorded immediately before BP measurements were analyzed and expressed as % oxygenated hemoglobin (SmO2 = 100 × oxygenated hemoglobin ÷ (oxygenated hemoglobin + deoxygenated hemoglobin)).

### 2.6 Heart rate variability

Heart rate variability (HRV) was measured to assess the autonomic nervous system function during the pre-test, post-test, and 24-h post-test measurements. While the participants resting in a supine position, electrocardiogram standard limb leads (SE-1515, Edan Instruments Inc., Shenzhen, China) were placed on the torso, and RR intervals were measured for 15 min in a supine position. Subsequently, power spectral analysis of HRV was conducted using the Fast Fourier Transform and expressed as the ratio of the low frequency (0.04–0.15 Hz) to high frequency (0.15–0.4 Hz) (LF/HF ratio) to determine an overall balance between the sympathetic and parasympathetic activities.

### 2.7 Baroreflex sensitivity

Baroreflex sensitivity (BRS) was measured using a finger photoplethysmography (Finometer Pro, Finapres Medical Systems B.V., Amsterdam, Netherlands) and the head-up tilt test (HUTT) during the pre-test, post-test, and 24-h post-test measurements. The participants were first rested in a supine position on a motorized tilt table (Multi Motorized Inversion Machine, Topspo, Gwangju, Republic of Korea) for 15 min followed by a head-up tilt to 50° for 15 min. During the test, beat-to-beat BP and pulse intervals were continuously measured, and the data from the 14th - 15th minute in each position were extracted for data analysis. BRS was calculated using Beatscope Easy (Finapres Medical Systems B.V., Amsterdam, Netherlands) through a time-domain cross-correlation method. This technique involved interpolating heartbeats using splines at a 1-s interval. A sliding correlation window, spanning 12 beats with 5 beats of overlap, was employed to compute the cross-correlation between BP and pulse interval. Various delays (0–5 s) were tested, and the delay with the highest correlation was selected. A BRS estimate was considered valid if the coefficient of determination ( $r^{2}$ ) was significant (*p* < 0.05) (Westerhof et al., 2004).

### 2.8 24-h ambulatory blood pressure

Ambulatory blood pressure (ABP) was measured to assess PEH in a 24-h window. After the completion of each experimental participation, the participants were equipped with an ABP monitor (ABPM50, Contec Medical System Co., Ltd., Qinhuangdao, China) for 24 h. The monitoring time window extended from approximately 12 p.m. on the experimental day to 12 p.m. the following day. ABP was measured at 30-min intervals during waking hours and at 60-min intervals during sleep hours. During the monitoring period, the participants were instructed to fall asleep no later than 23:00, in consideration of the experiment time (07:00–12:00), and to maintain a lifestyle similar to their daily routine, while refraining from alcohol consumption and strenuous physical activity. They were also instructed to record a daily activity log such as the time spent at work/home and hours of sleep. ABP data were analyzed to derive mean awake and sleep BP values.

### 2.9 Statistical analyses

All data in this study were analyzed using SPSS (Ver. 26, IBM Corp., New York, United States) and presented as mean and standard deviation. Two-way repeated measures ANOVA was used to compare dependent variables between CON and IPC. When a significant F-value, adjusted for sphericity using Greenhouse-Geisser correction, was identified, one-way repeated measures ANOVA followed by *post hoc* pairwise comparison with Bonferroni correction was conducted to compare variables across the time course and conditions at each time point, respectively. The significance level of all statistical analyses was set at α = 0.05.

## 3 Results

All participants met the eligibility criteria, with six participants having elevated SBP (120–129 mmHg), and six participants categorized as having stage 1 hypertension (5 with SBP ≥ 130 mmHg, 1 with DBP ≥ 80 mmHg) (Table 1). All participants completed the study protocols without any adverse effects.

**TABLE 1:** Summary of participant characteristics (*n* = 12).

Variables	Subjects (*n* = 12)
Age (years)	24 ± 2.6
Height (cm)	178.9 ± 5.5
Weight (kg)	79.4 ± 9.0
Body mass index (kg/m^2^)	24.7 ± 2.1
Systolic blood pressure (mmHg)	131.2 ± 5.9
Diastolic blood pressure (mmHg)	77.3 ± 4.8
Resting heart rate (bpm)	77.3 ± 15.0
Peak oxygen uptake (mL/kg/min)	35.1 ± 5.9
Peak exercise intensity (watts)	124.2 ± 13.9

Note. Values are mean ± standard deviation.

### 3.1 Hemodynamic responses during exercise and recovery

**FIGURE 2:**
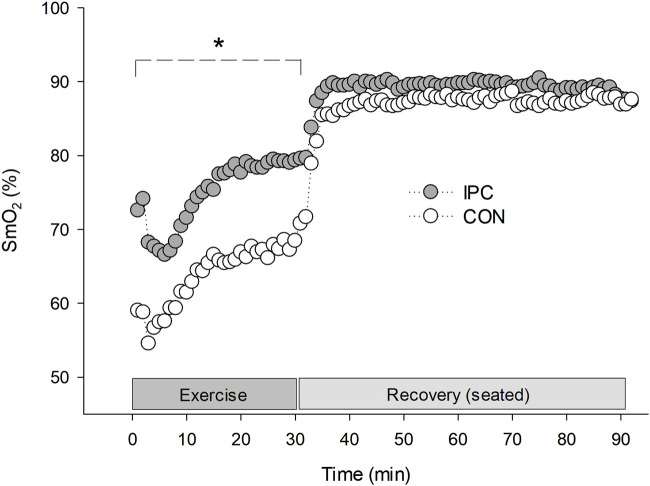
Rectus femoris muscle oxygen saturation during exercise and 1-h recovery. Values are presented as a group mean (n = 12). * Significantly different from control (*p* < 0.05).

Hemodynamic responses during exercise and recovery are shown in Table 2 and Figure 2. Although no significant interaction was detected, a significant main effect (F = 71.562, *p* = 0.001) was observed for SBP throughout the recovery in both conditions, indicating a noticeable PEH up to 60 min in IPC compared to CON, where SBP returned to baseline values beyond 36 min of recovery. Otherwise, no significant difference was found for DBP, MAP, and HR during exercise and 1-h recovery between the conditions.

**TABLE 2:** Hemodynamic responses during and after exercise (*n* = 12).

		During exercise				Post-ex rest					F	*p*
		0 min	0 min	12 min	21 min	30 min	P12 min	P24 min	P36 min	P48 min			P60 min
SBP (mmHg)	CON	128.1 ± 7.6	168.9 ± 17.4	162.8 ± 12.9	165.1 ± 19.9	124.7 ± 4.6	122.0 ± 11.2	120.8 ± 12.4	121.7 ± 12.5	124.7 ± 11.1	0.388	C: 0.463 T: 0.001 C×T: 0.760
	IPC	129.2 ± 7.6	163.3 ± 13.9	164.1 ± 21.9	158.4 ± 20.1	123.3 ± 13.9	121.8 ± 12.8	120.2 ± 7.4	120.4 ± 9.6	122.2 ± 13.7		
DBP (mmHg)	CON	76.9 ± 14.0	64.0 ± 12.1	64.67 ± 10.4	66.7 ± 14.8	74.0 ± 9.2	77.1 ± 9.1	76.2 ± 6.8	76.0 ± 8.9	78.3 ± 9.8	1.392	C: 0.14 T: 0.01 C×T: 0.259
	IPC	78.7 ± 12.2	74.7 ± 9.7	68.9 ± 8.8	66.6 ± 11.8	76.1 ± 6.6	78.9 ± 6.5	79.9 ± 7.0	80.7 ± 8.7	80.9 ± 6.7		
MAP (mmHg)	CON	93.9 ± 11.1	98.9 ± 11.8	97.37 ± 8.9	99.2 ± 10.3	90.9 ± 6.5	92.1 ± 9.5	91.1 ± 8.0	91.2 ± 9.1	93.8 ± 9.7	0.737	C: 0.416 T: 0.016 C×T: 0.583
	IPC	95.6 ± 9.9	104.22 ± 9.8	100.63 ± 10.0	97.2 ± 12.4	91.2 ± 6.9	93.2 ± 6.9	93.3 ± 6.6	93.9 ± 8.4	94.7 ± 8.3		
HR (BPM)	CON	79.7 ± 10.8	139.1 ± 10.2	145.22 ± 15.1	150.4 ± 15.8	90.0 ± 14.7	85.2 ± 11.6	79.4 ± 12.9	76.1 ± 13.7	74.9 ± 15.4	1.545	C: 0.271 T: 0.001 C×T: 0.211
	IPC	74.9 ± 7.6	139.1 ± 14.5	145.22 ± 15.1	147.2 ± 17.3	88.6 ± 10.9	77.0 ± 11.9	75.6 ± 12.2	73.9 ± 11.6	72.9 ± 8.9		

Values are mean ± standard deviation (n = 12). SBP, systolic blood pressure; DBP, diastolic blood pressure; MAP, mean arterial pressure; HR, heart rate; C, main effect of condition; T, main effect of time; C×T, interaction.

A significant interaction was observed for SmO_2_ between the conditions (F = 6.399, *p* = 0.004). The difference was noted as a significantly elevated SmO_2_ throughout the exercise in IPC compared to CON. The enhanced oxygenation trend in IPC persisted above CON during the recovery, despite statistical significance not being achieved.

### 3.2 Heart rate variability and baroreflex sensitivity

Table 3 demonstrates HRV and BRS responses to the head-up tilt test between IPC and CON. No significant interaction was found for HRV between the conditions. Regarding BRS, there was also no significant difference in supine BRS, despite the improved responsiveness with IPC. However, a significant interaction was found in tilted BRS between the conditions. This difference was characterized by significantly reduced BRS after exercise (*p* = 0.015) extending to 24h-post measurements (*p* = 0.018) in CON while IPC preserved BRS similar to pre-exercise values.

**TABLE 3:** Summary of heart rate variability and baroreflex sensitivity during head-up tilt test.

		CON			IPC			F	*p*
		Pre	Pre	Post	24h-post	Pre	Post			24h-post
LF/HF ratio		1.8 ± 1.5	1.4 ± 0.4	2.0 ± 0.7	1.7 ± 1.9	1.3 ± 0.9	1.7 ± 1.1	0.225	C: 0.612 T: 0.218 C×T: 0.8
BRS (ms/mmHg)	Supine (0°)	16.8 ± 8.7	20.6 ± 15.0	14.1 ± 6.3	18.8 ± 11.3	33.2 ± 18.3	25.5 ± 13.6	1.767	C: 0.097 T: 0.033 C×T: 0.209
	Standing (50°)	10.8 ± 4.7	8.7 ± 4.4	7.1 ± 2.4	9.9 ± 2.4	12.3 ± 3.9*	11.2 ± 4.6*	4.024	C: 0.002 T: 0.351 C×T: 0.047

Values are mean ± standard deviation (n = 12). LF, low frequency; HF, high frequency; BRS, baroreflex sensitivity; C, main effect of condition; T, main effect of time; C×T, interaction. * Significantly different from control (*p* < 0.05).

### 3.3 24-h ambulatory blood pressure

**FIGURE 3:**
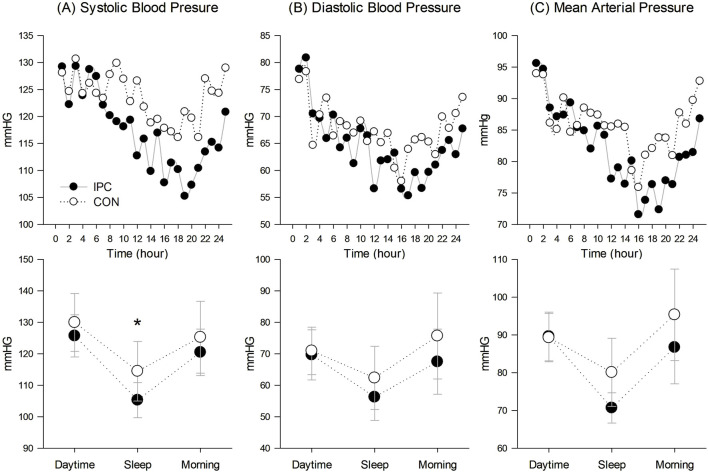
24-hour ambulatory blood pressure responses. **(A)** Systolic blood pressure responses in 24-hour trend and in daytime, sleep, and morning, **(B)** Diastolic blood pressure responses in 24-hour trend and in daytime, sleep, and morning, and **(C)** Mean arterial pressure responses in 24-hour trend and in daytime, sleep, and morning. Values are presented as a group mean for the 24-hour trend and mean with standard deviation for the breakdown of values in daytime, sleep, and morning (*n* = 12). * Significantly different from control (*p* < 0.05).

Table 4 and Figure 3 demonstrate 24-h ABP responses between IPC and CON. A significant interaction effect between condition and time was observed for SBP indicating a notable reduction in mean sleep SBP (*p* = 0.003) in IPC compared to CON. However, this reduction was not significant for awake SBP (*p* = 0.095). No significant interaction was found for either awake or sleep DBP and MAP between the two conditions.

**TABLE 4:** Summary of 24-h ambulatory mean awake and sleep blood pressure.

	CON		IPC		F	*p*
	Awake	Sleep	Awake	Sleep		
SBP (mmHg)	127.4 ± 7.2	114.8 ± 9.3	122.9 ± 8.0	103.1 ± 4.2*	5.574	C: 0.006 T: 0.001 C×T: 0.046
DBP (mmHg)	72.8 ± 5.2	62.3 ± 10.1	69.7 ± 7.4	57.1 ± 6.9	0.458	C: 0.140 T: 0.001 C×T: 0.517
MAP (mmHg)	90.5 ± 5.7	78.1 ± 12.3	88.1 ± 6.9	70.5 ± 4.1	2.042	C: 0.103 T: 0.001 C×T: 0.191

Values are mean ± standard deviation (n = 12). C, main effect of condition; T, main effect of time; C×T, interaction. * Significantly different from control (*p* < 0.05).

## 4 Discussion

To the best of our knowledge, this is the first study to investigate the efficacy of IPC combined with aerobic exercise on PEH in pre- or hypertensive men, especially being monitored for 24-h period. The present results indicate that IPC applied before an acute bout of aerobic exercise leads to a significant reduction of ambulatory SBP especially during sleep compared to the aerobic exercise alone. Such an observed SBP reduction was marked by a maintained BRS, persisting up to 24 h post-measurements in contrast to a sustained decrease in standing BRS after exercise in CON. These findings align with our hypothesis that IPC is likely to elicit a more pronounced PEH supplementary to aerobic exercise, with enhanced BRS being a contributing factor to this intensified PEH.

A single bout of aerobic exercise has been demonstrated to induce temporal BP reduction, and this response appears in both healthy individuals and patients with cardiovascular risks such as hypertension and diabetes (Keese et al., 2011; Ciolac et al., 2009; de Morais et al., 2015; Ferrari et al., 2017). Previous findings also showed that the greater the exercise intensity, the greater the BP reduction effect especially in hypertensive individuals (Quinn, 2000; Hagberg et al., 1987; Jones et al., 2008). The rapid BP rise response during exercise not only acts as a predictor of hypertension, but also can spark acute myocardial infarction (Giri et al., 1999), low-to-moderate intensity exercise is usually recommended to prevent an exaggerated increase in BP during exercise. Interestingly, this level of intensity for targeting hypertensive was maintained PEH for 90 min in hypertensives individuals, as opposed to the normotensive individuals who did not show PEH at all (Cléroux et al., 1992). Although the exercise protocol in the present study was similar to that of a previous study (50% VO_peak_), the CON condition only maintained a significant SBP reduction up to 36 min post-exercise (Table 2). This may be due to the non-severe high BP levels of out subjects. On the other hand, there has been evidence that BP lowering effect of acute IPC could appear in both hypertensive rats and patients including peripheral artery disease, angina pectoris, and obese (Farkašová et al., 2023; Kuusik et al., 2019; Zagidullin et al., 2016; Jang et al., 2023). Additionally, the combination of IPC and resistance exercise significantly decreased SBP, DBP, and MBP for up to 60 min post-exercise compared to resistance exercise alone in normotensives (Panza et al., 2020). Although the type of exercise was different, our results presented a similar pattern of PEH reduction pattern. The combination of IPC and aerobic exercise maintained a significant SBP reduction compared to resting values from post-60 min onward in the laboratory setting (Table 2).

The observed exaggeration of IPC-induced PEH is deemed to enhance physiological cascades responsible for inducing PEH. In addition to the activation of arterial baroreceptor in response to increased systemic arterial pressure leading to a decrease in sympathetic outflow and an increase in vagal cardioinhibitory outflow (de Paula et al., 2019), these mechanisms may also involve reduced peripheral vascular resistance, cardiac contractility, and venous return (La Rovere, Pinna and Raczak, 2008). Additionally, vasodilators released by acute exercise (Pescatello et al., 2004) as well as by the humoral and neural pathways of IPC (Hausenloy and Yellon, 2008) can contribute to these mechanisms. For instance, the increase in blood flow following exercise or release of occlusion occurs through the stimulation of endothelium by shear stress. This stimulation leads to the activation of endothelial nitric oxide (NO) synthesis mediated by protein kinase C and calmodulin. Subsequently, increased NO diffuses to vascular smooth muscle cells and activates cyclic guanosine monophosphate-dependent protein kinase G. It provokes the phosphorylation of myosin light chain kinase, thereby causing vasodilation. In fact, Crisafulli (2006) suggested the possibility that exercise and IPC can be alternatives to each other for cardioprotection since they share common pathway such as increased NO production via shear stress and activation of protein kinase C. Meanwhile, aerobic exercise with blood flow restriction intervention was effective in amplifying the PEH, but BP during exercise was higher than that of aerobic exercise alone (Barili et al., 2018). On the contrary, our finding confirmed that aerobic exercise following IPC showed no significant difference in BP during exercise compared to aerobic exercise alone, along with PEH reinforcement. This implies that IPC may be a safer option than blood flow restriction.

BP fluctuates between waking and sleeping according to the circadian rhythm, typically decreasing by over 10% during sleep compared to waking. Particularly, sleep BP can only be accurately assessed through ABP monitoring, and elevated sleep BP is significantly associated with an unfavorable prognosis (Verdecchia et al., 1994). The present results (Table 4; Figure 3) demonstrate a notable interaction in ambulatory SBP, whiles awake SBP, comparing daytime and morning BP, exhibited a non-significant trend (*p* = 0.095). These findings are consistent with Cartner’s study, which showed a significant BP reduction during sleep, but not in awake BP in pre-hypertensive individuals (Cartner, 2011). We intended to conduct the experiment in the morning (07:00–12:00), and thereafter instructed the subjects to continue with their usual daily activities. Somers et al. (1991) suggested that PEH is likely to dissipate due to these everyday activities, which could have potentially masked the PEH effect observed in a laboratory setting. Therefore, the lack of significant differences in awake BP between the conditions are likely due to the influence of daily activities, potentially masking the PEH effect observed in a controlled setting, which may however, be more evident during sleep. This speculation is supported by our findings on sleep SBP. According to sleep BP classification, expressed as sleep-to-awake BP ratio (Krzych and Bochenek, 2013), our subjects in the CON were categorized as non-dippers (0.9), whereas those in the IPC were classified as the dippers (0.83), suggesting a potentially lower cardiovascular risk. A reduction in sleep SBP, a potent risk factor for cardiovascular disease, is significantly linked to a decreased likelihood of cardiovascular disease-related morbidity and mortality (Hermida et al., 2018). Despite prior research suggesting that morning exercise reduces sleep SBP more effectively than afternoon exercise (Fairbrother et al., 2014; Cartner, 2011), the CON condition in our study presented non-significant effects, possibly due to lower exercise intensity compared to previous studies. IPC has been shown to improve sleep quality (Wu et al., 2023) and contribute to ABP reduction (Tong et al., 2019; Guo et al., 2023). In summary, unlike aerobic exercise alone, the addition of IPC to aerobic exercise decreased sleep SBP, and a declining trend was observed in awake systolic BP. Considering that the PEH reduction with IPC was maintained throughout the laboratory measurements, it can be assumed that sleep SBP reduction was originated from an extension of PEH. However, due to the absence of baseline ABP measurements, we could not determine the comparative effectiveness of aerobic exercise alone on baseline sleep SBP.

Patients with hypertension may experience increased vasoconstriction in active muscles due to impaired functional sympatholysis during exercise. This can lead to a decrease in SmO_2_ in exercising muscles or necessitate an excessive increase in BP to compensate (Dipla et al., 2017). However, the present results showed that IPC improved SmO_2_ during exercise without compensatory BP rise compared to the CON condition, suggesting improved oxygen utilization in response to the same metabolic demand (Figure 2). Furthermore, the higher SmO_2_ level at 0 min in comparison to CON indicates that the elevated SmO_2_ level during exercise has been maintained since before the exercise session began. This finding is consistent with our previous results and another study, which showed that IPC remains relatively high in reactive hyperemia and persists for several minutes even during rest in both obese individuals (Jang et al., 2023) and those with lower limb occlusive arterial disease (Fudickar et al., 2014). High SmO_2_ during exercise may result from either active vasodilation of the exercising muscles (Saltin and Mortensen, 2012) or suppressed vasoconstriction induced by sympathetic overactivation (Thomas and Segal, 2004), or a combination of both factors. In fact, we have previously confirmed that IPC inhibits sympathetic hyperactivation (Jang et al., 2023), and others also reported IPC-mediated improvement in NO bioavailability, which leads to vasodilation of exercising muscles (Lambert et al., 2016). Unfortunately, our subjects did not present a significant inhibition of sympathetic activation when monitored by HRV in this study. However, increased muscle blood flow, and consequently increased SmO_2_, is one of the possible contributors to PEH. Jeffries et al. (2018) and Jones et al. (2014) described that ameliorated microcirculation by repeated IPC kept up beyond 72 h together with a significant BP drop during the rest. This additional IPC-induced vasodilation effect mediated by endothelium dependent and/or independent factors might intensify the reduction in the total peripheral resistance (afterload) while increasing systemic blood flow shown as improved SmO_2_ in the present results.

The arterial baroreflex, a crucial factor in the neural regulation of cardiovascular system (La Rovere et al., 2008) was shown to be impaired in hypertension compared to normotension in both animals and humans (Ricksten and Thoren, 1981; Miyajima, 1986). In patients with autonomic dysfunction, both aerobic and strength exercises were effective for improving cardiovagal BRS with the former having a greater effect post-exercise (de Paula et al., 2019). In a similar context, IPC is recognized as a method to prevent the deterioration of BRS associated with prolonged ischemia (Babai et al., 2002). Notably, evidence suggests that IPC enhances post-exercise cardiac vagal reactivation (Sabino-Carvalho et al., 2019). Consistent with this, the present results shows that the combination of IPC and aerobic exercise significantly improved cardiovagal BRS in the standing position (Table 3). Therefore, it is plausible that the improvement in BRS induced by IPC contributed to the greater and more prolonged PEH observed compared to aerobic exercise alone. However, unlike BRS, no significant interaction was found in the LF/HF ratio (Table 3). We measured the LF/HF ratio only in the supine position, aligning with the supine BRS results, which also showed no significant effect. Considering that postural changes, particularly standing, can affect the LF/HF ratio in individuals with borderline hypertension (Duprez et al., 1995), the measurement of the LF/HF ratio only in the supine position could be a limitation of our study. Alternatively, it is possible that in our hypertensive subjects, another mechanism identified by the previous study where vasodilation-mediated PEH occurs alongside sympathetic activation (Hara and Floras, 1995), may have played a more dominant role in reducing blood pressure. This mechanism, independent of the inhibition of sympathetic activation as indicated by the LF/HF ratio, suggests there may be additional factors influencing PEH in pre- or hypertensive men.

Our study has several limitations. The sample size, while sufficient to detect meaningful within-group differences and treatment effects, limits the generalizability of the findings to broader populations. We selected 12 participants to ensure manageable data collection and adherence to the study protocol, which was also based on previous studies examining vascular function and blood pressure outcomes in similar populations (Tong et al., 2019; Kim et al., 2018; 2022). However, larger sample sizes are needed for future studies to confirm our results across more diverse populations.

Additionally, due to potential hormonal fluctuations affecting vascular function, we excluded female participants. This decision was in line with prior research indicating that estrogen, especially during the follicular phase of the menstrual cycle, enhances vasodilation and endothelial function through increased nitric oxide availability (Koh, 2002). However, excluding women reduces the applicability of our findings to the general population. Including both genders in future research would enhance the generalizability of these results.

Another limitation is that the study design is the use of only one familiarization session for participants. The previous research on PEH and related vascular outcomes has highlighted the importance of multiple familiarization sessions to ensure participants are fully adapted to the testing protocol, thus minimizing variability in physiological responses (De Brito et al., 2019). A single familiarization session might not be enough for participants to become fully acclimated, potentially introducing variability in exercise and IPC interventions. While more familiarization sessions could have provided consistency of responses and accurate interpretation of outcomes, we aimed to reduce the burden on subjects, who were already required to attend five visits for the present experiment. This decision was made to enhance participants’ compliance and minimize fatigue associated with multiple sessions.

Furthermore, the study would have benefited from the inclusion of an IPC-only trial or true time control without exercise to better determine the magnitude of the effect on blood pressure. By not including an IPC-only group, it becomes difficult to fully discern whether the observed antihypertensive effects were due to the combined intervention or primarily driven by IPC alone. Including this comparison could have helped quantify the relative contribution of IPC versus aerobic exercise in reducing blood pressure, especially considering prior studies have shown IPC to have significant effects on blood pressure independent of exercise (Madias, 2011; Zagidullin et al., 2016; Kuusik et al., 2019; Jang et al., 2023).

## 5 Conclusion

In conclusion, combining IPC with aerobic exercise extended the PEH effects of aerobic exercise over an extended period in pre- or hypertensive men. Although this reduction in blood pressure was briefly masked during waking hours by daily activities, it contributed to a significant decrease in SBP during sleep. The sustained or intensified blood pressure reduction may be linked to the significant improvement in BRS observed up to 24 h post-exercise or the enhanced peripheral vasodilation evidenced by SmO_2_, which increased significantly during exercise compared to the control condition. Therefore, applying IPC before aerobic exercise could be a valuable approach, offering additional benefits over aerobic exercise alone, such as more efficient oxygen delivery to the working muscles and prolonged PEH. Further research is needed to explore the long-term effects together with invasive methods to investigate the underlying mechanisms.

## Data Availability

The original contributions presented in the study are included in the article/supplementary material, further inquiries can be directed to the corresponding author.
